# Network-based stratification analysis of 13 major cancer types using mutations in panels of cancer genes

**DOI:** 10.1186/1471-2164-16-S7-S7

**Published:** 2015-06-11

**Authors:** Xue Zhong, Hushan Yang, Shuyang Zhao, Yu Shyr, Bingshan Li

**Affiliations:** 1 Department of Biostatistics, Vanderbilt University, Nashville, TN, USA; 2 Center for Quantitative Sciences, Vanderbilt University, Nashville, TN, USA; 3 Department of Medical Oncology, Thomas Jefferson University, Philadelphia, PA, USA; 4 Department of Statistics, Middle Tennessee State University, Murfreesboro, TN, USA; 5 Cancer Biology, Vanderbilt University, Nashville, TN, USA; 6 Molecular Physiology and Biophysics, Vanderbilt University, Nashville, TN, USA

**Keywords:** tumor somatic mutations, networks, subtyping, network-based-stratification

## Abstract

**Background:**

Cancers are complex diseases with heterogeneous genetic causes and clinical outcomes. It is critical to classify patients into subtypes and associate the subtypes with clinical outcomes for better prognosis and treatment. Large-scale studies have comprehensively identified somatic mutations across multiple tumor types, providing rich datasets for classifying patients based on genomic mutations. One challenge associated with this task is that mutations are rarely shared across patients. Network-based stratification (NBS) approaches have been proposed to overcome this challenge and used to classify tumors based on exome-level mutations. In routine research and clinical applications, however, usually only a small panel of pre-selected genes is screened for mutations. It is unknown whether such small panels are effective in classifying patients into clinically meaningful subtypes.

**Results:**

In this study, we applied NBS to 13 major cancer types with exome-level mutation data and compared the classification based on the full exome data with those focusing only on small sets of genes. Specifically, we investigated three panels, FoundationOne (240 genes), PanCan (127 genes) and TruSeq (48 genes). We showed that small panels not only are effective in clustering tumors but also often outperform full exome data for most cancer types. We further associated subtypes with clinical data and identified 5 tumor types (CRC-Colorectal carcinoma, HNSC-Head and neck squamous cell carcinoma, KIRC-Kidney renal clear cell carcinoma, LUAD-Lung adenocarcinoma and UCEC-Uterine corpus endometrial carcinoma) whose subtypes are significantly associated with overall survival, all based on small panels.

**Conclusion:**

Our analyses indicate that effective patient subtyping can be carried out using mutations detected in smaller gene panels, probably due to the enrichment of clinically important genes in such panels.

## Background

Cancers are complex diseases with highly heterogeneous causes and clinical outcomes. At the molecular level histologically and clinically similar patients often exhibit drastically distinct genomic aberrations. Large-scale studies such as The Cancer Genome Atlas (TCGA) have comprehensively cataloged multi-layered genomic aberrations in multiple cancers [1,2]. Identification of clinically meaningful subtypes of tumors based on molecular patterns is critical to provide insights into the biological mechanisms of tumor progression and to guide better treatment and prognosis. Most previous studies of tumor classification utilized messenger RNA (mRNA) expression [3-5], which often yielded subtypes that are not highly predictive of clinical outcomes [1,6]. On the other hand, somatic mutations, which often disrupt the function of mutated genes, provide insights not only to the mechanisms of tumorigenesis and progression but also the candidates for targeted therapy [7-9]. Therefore classification of patients based on somatic mutations may provide more effective clinical guidance. However, mutations are rarely shared across patients [10,11] so that the similarity between tumors cannot be directly measured based on mutated genes. Network-based stratification (NBS) was recently proposed to overcome this challenge by leveraging information provided in protein-protein interaction networks (PPI) [12]. Briefly, NBS uses label propagation on PPI to assign higher values to non-mutated genes that are closer to genes (in PPI) that harbor mutations. This guilt-by-association principle governed by genetic networks has many applications for biological discovery utilizing prior knowledge [13,14]. For somatic mutations in genes, this principle fits well with the underlying biology: driver genes are often interacting directly or indirectly in common pathways and mutations in different genes in the same pathway are likely to cause genetically similar tumors [11,15-18]. NBS has been applied on several cancers using exome-level mutation data and showed improved association of subtypes with clinical outcomes than using mRNA data [12]. In general NBS provides a unified framework to further investigate tumor subtyping by integrating somatic mutations with biological networks.

Exome-level mutations were used in previous NBS analyses. In routine research and clinical application, instead of exome sequencing, a viable cost-effective alternative is to screen mutations in a panel of pre-selected cancer genes [19,20]. It is unknown whether such small panels are effective in classifying patients into clinically meaningful subtypes. Although exome sequencing provides comprehensive characterization of coding mutations, it is likely that a large portion of mutations are passengers, as it was estimated that few mutations in a patient are drivers (e.g. ranging from 2 to 8) [16,21]. Such passengers, if included in the analysis, may obscure clinically and biologically important mutations. On the other hand, gene panels are usually designed to include known driver genes or genes involved in important pathways, resulting in highly enriched signal vs. noise. We reasoned that a panel of cancer genes, although likely that some important genes were not included, may provide adequate mutation information for effective subtyping. It is an important issue to investigate since ultimately it is only practical to routinely screen a panel of genes in clinical settings.

In this study, we set out to evaluate the effectiveness of various gene panels on classifying tumors into clinically meaningful subtypes. Specifically, we collected three panels for evaluation, representing different numbers of genes: FoundationOne, PanCan and TruSeq. Briefly, FoundationOne panel includes 240 genes; PanCan was derived from 12 cancer types analyzed by TCGA and includes 127 genes [16]; TruSeq was developed by Illumina and includes 48 genes. We applied the NBS approach to 13 major cancer types with a total of ~4000 solid tumor samples profiled by exome-sequencing, using the full exome-level mutation data (termed "Full" dataset) as well as the three cancer panels.

## Materials & methods

### Cancer gene panels and mutation data

**Figure 1:**
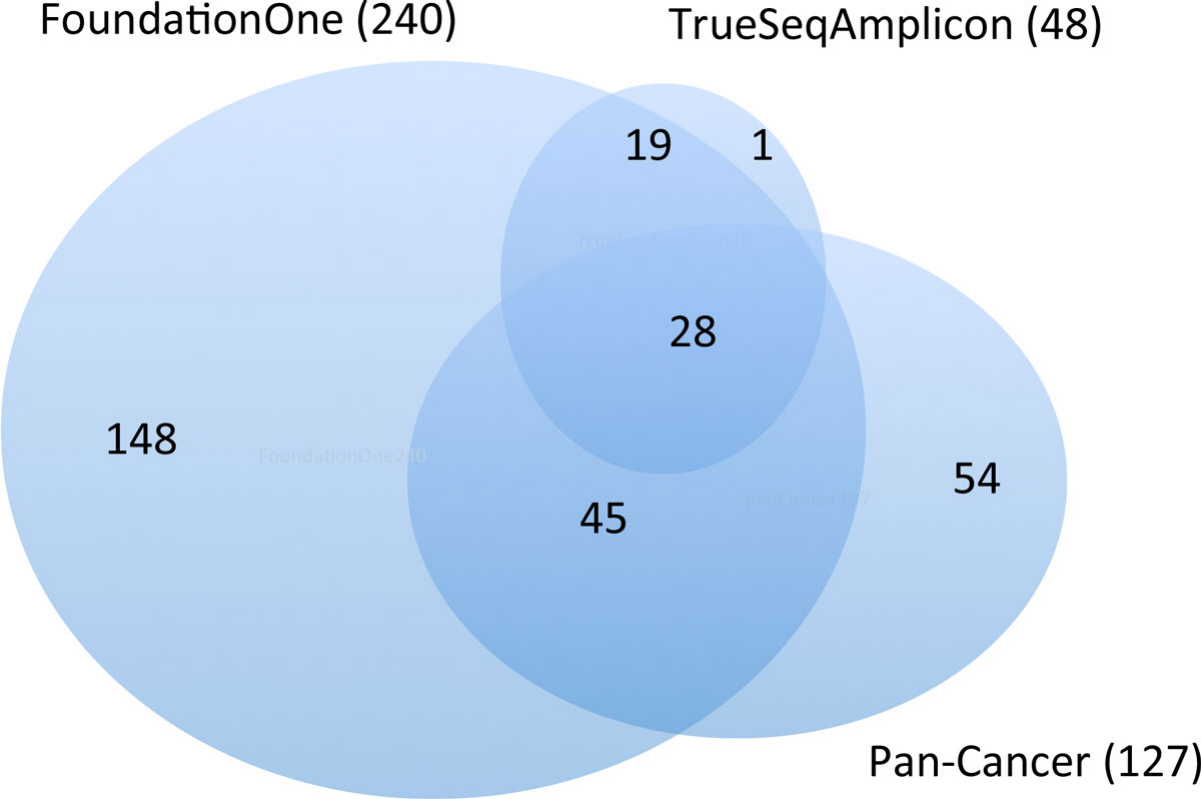
**Relationship of the gene lists in the three small panels**.

Three gene panels were investigated: FoundationOne (June 2014 version) (http://foundationone.com/genelist1.php), PanCan 127-gene panel [16] and TrueSeq (http://www.illumina.com/products/truseq_amplicon_cancer_panel.ilmn). The relationship of the gene lists in the three panels is illustrated in Figure 1.

We initially collected mutation data on ~ 4700 solid tumors of 21 major cancer types that were profiled by exome-sequencing and investigated previously [10] (http://cancergenome.broadinstitute.org). In this study, we focus on mutations that are non-synonymous, occurred on splice sites or stop codons (termed "functional" mutations). In contrast, the non-functional mutations refer to the synonymous mutations and mutations in intronic or intergenic regions. Samples with too few mutations were treated as outliers and excluded from analysis. Specifically, samples with no mutations in gene panels or fewer than 6 functional mutations in exomes were discarded. Several cancer types, such as CARC, CLL, DLBCL and LAML that are low in mutation rates have only dozens of samples after filtering. These cancer types were excluded from further analyses. Due to the extreme molecular similarity between colon and rectal cancers [22], we combined these two into a single CRC dataset. Finally, we are left with 13 cancer types with a total of ~4000 samples (Table 1) (See Table S2 in Additional file for all 21 cancer types). For each sample we coded a gene as 1 if it has at least one functional mutation and zero otherwise. The mutation profile per patient is represented by a vector of genes marked with 1's (mutated) or 0's (not mutated).

**Table 1:** Sample sizes of 13 tumor types before and after filtering to ensure sufficient mutations per sample and total samples.

Cancer^a,b^	Sample size	Full (# mutations ≥ 6)	FoundationOne (# mutations ≥ 1)	PanCan (# mutations ≥ 1)	TrueSeq (# mutations ≥1)
**BLCA**	99	99 (100%)	97(98%)	95 (96%)	84 (85%)

**BRCA**	887	849 (96%)	661 (75%)	647 (73%)	528 (60%)

**CRC**	233	233 (100%)	227 (97%)	226 (97%)	219 (94%)

**ESO**	140	140 (100%)	133 (95%)	125 (89%)	112 (80%)

**GBM**	291	288 (99%)	247 (85%)	237 (81%)	199 (68%)

**HNSC**	384	372 (97%)	357 (93%)	347 (90%)	303 (79%)

**KIRC**	417	414 (99%)	328 (79%)	310 (74%)	220 (53%)

**LUAD**	398	391 (98%)	372 (93%)	359 (90%)	322 (81%)

**LUSC**	176	176 (100%)	176 (100%)	175 (99%)	158 (90%)

**MEL**	118	118 (100%)	117 (99%)	113 (96%)	112 (95%)

**MM**	204	200 (98%)	157 (77%)	146 (72%)	121(59%)

**OV**	316	313 (99%)	276 (87%)	281 (89%)	238 (75%)

**UCEC**	247	247 (100%)	245 (%99)	242 (98%)	229 (93%)

**Total**	4143	4008			

^a^BLCA-Bladder urothelial carcinoma; BRCA-Breast invasive carcinoma; CRC-Colorectal carcinoma; ESO-Esophageal adenocarcinoma; GBM-Glioblastoma multiforme; HNSC-Head and neck squamous cell carcinoma; KIRC-Kidney renal clear cell carcinoma; LUAD-Lung adenocarcinoma; LUSC-Lung squamous cell carcinoma; MEL-Melanoma; MM-Multiple myeloma; OV-Ovarian serous cystadenocarcinoma; UCEC-Uterine corpus endometrial carcinoma

^b^Cancer types with survival data: BLCA, BRCA, CRC, ESO, GBM, HNSC, KIRC, LUAD, LUSC, OV,UCEC.

### Gene interaction network

HumanNet [23] v.1 (http://www.functionalnet.org/humannet/download.html) with the top 10 percent most confident edges were used as the gene-to-gene interaction network. Mutations were mapped to genes on this network for label propagation (see below). This gene interaction network was also transformed into a *k*-nearest-neighbors graph by connecting vertices (i.e. genes) *v_i _*and *v_j _*if *v_i _*is among the *k*-nearest neighbors of *v_j _*or if *v_j _*is among the *k*-nearest neighbors of *v_i_*. The result is a symmetric connectivity matrix, which was used to derive the graph Laplacian in network-regularized NMF (see below).

### Network propagation

Let *n *be the number of patients, *m *be the number of genes, F_0 _be the initial gene × patient matrix (m*×n *matrix), and A be the symmetric adjacency matrix representing gene-to-gene interaction network (*m×m *matrix). The network propagation process is carried out by the following iterative algorithm [12]:

(1) $$F_{t+1}=a\cdot A\cdot F_{t}+\left(1-a\right)\cdot F_{0}$$

We set a=0.7 as previously described [12]. The propagation function was run iteratively until F_t _converges (|F_t+1 _-F_t_| < 0.001). Following the propagation, quantile normalization was applied to F_t _to ensure each patient follows the same distribution for the smoothed mutation profile. We use F to denote the final normalized and smoothed mutation matrix.

### Network-regularized NMF

Non-negative matrix factorization (NMF) aims to decompose a matrix into two lower rank non-negative matrices whose product can well approximate the original matrix [24]. We applied network-regularized NMF to constrain NMF to respect the structure of the underlying gene interaction network as previously described [25]. The objective is to minimize the following function:

(2) $$min_{W,H>0}||F-WH|{|}_{F}^{2}+\lambda \;trace\left(W^{t}LW\right)$$

where $||\cdot |{|}_{F}$ denotes the matrix Frobenius norm, *W *is an *m *by *K *matrix and *H *is a *K *by n matrix, with entries in both *W *and *H *non-negative. *W *is a collection of basis vectors or "metagenes", and *H *contains the loading of the basis. The value *K *controls the dimension reduction, and we used *K *= 3,4,5,6 in this study. *L *is the graph Laplacian of a *k*-nearest-neighbor network. We chose *k *= 11 as previously described [12]. λ is the regularization parameter and the value was set as 200, which is on the same scale as previously described [25]. The iterative algorithm proposed in Cai et al [25] was used to find solutions *W *and *H*. The iteration was run until the objective function converges (|F_t+1 _-F_t _| < 0.1).

### Consensus clustering

In order to achieve robust clustering, we used consensus clustering [26] to generate final clustering of patients. Specifically, we ran network-regulated NMF using a random sample without replacement of 80% patients to construct a clustering, and repeated this process 500 times. The collection of 500 clustering results was used to construct the similarity matrix, which records the frequency with which each pair of patients was observed to share the same membership among all replicates. Hierarchical clustering with average linkage was generated based on the similarity matrix using the R "NMF" package. We used cophenetic correlation coefficient (ccc) to assess the dispersion of the consensus matrix as previously described [27] also using the R "NMF" package.

### Survival analysis

Survival analysis was performed using the R "survival" package. Kaplan-Meier survival curves were plotted for each NBS subtype and log-rank tests were performed to test the association of subtypes with survival. Fisher's exact tests were used to test the association of subtypes with tumor grade or stage.

## Results

### Clustering patterns

We examined the clustering patterns of 13 cancer types for each of the gene sets (the three panels plus the Full dataset) by applying the NBS approach. The clustering outcomes (*K *= 3, 4, 5, 6) for each of the 13 cancer types were displayed in Supplemental Fig. S1-S13 (Additional file). Overall, we observed different clustering patterns across four sets of genes for a given cancer type and a rank *K*. For most cases, the smallest panel (TrueSeq) produced the clearest stratification while for the Full data the stratifications are less clear (e.g., ESO, GBM, KIRC, MM). In a few cases similar patterns were observed for different gene panels (e.g., CRC, HNSC). FoundationOne and panCan panels often produced clusters of unbalanced sizes. Among the three panels, FoundationOne and PanCan tend to produce similar patterns (e.g., KIRC, HNSC, GBM, LUAD, MM). For a few cancers (e.g., LUSC, OV) all three panels generated similar patterns. For a fixed panel, the clustering outcome appears insensitive to *K *(e.g., BLCA, KIRC, HNSC, LUSC, MEL and MM). The goodness of the cluster separation was assessed using the cophenetic correlation coefficient (ccc) and for clusters that exhibit clear patterns the ccc values are over 0.99. Consistent with the visual impression, the TrueSeq panel in general gave the clearest separation (median of ccc = 0.988), followed by PanCan (median ccc = 0.982), FoundationOne (median ccc = 0.972) and the Full set (median cccc = 0.627).

### NBS subtypes associated with clinical data

**Figure 2:**
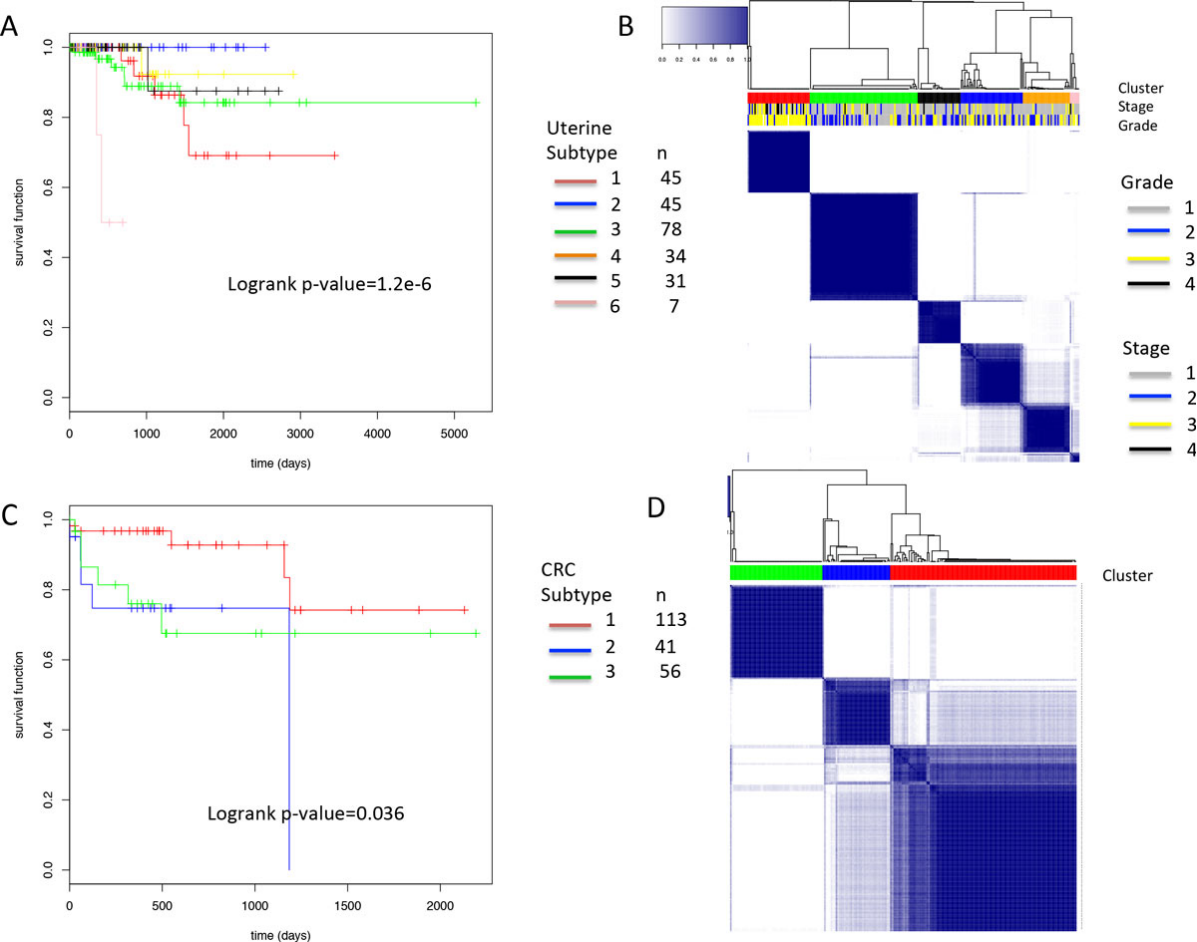
**Subtypes based on the TrueSeq panel and the association with survival for UCEC (A, B) and CRC (C, D)**.

We first investigated the association between clusters and survival and found that not all subtypes are associated with survival. Of the 11 cancer types with survival information available, 5 (CRC, HNSC, KIRC, LUAD and UCEC) showed significant association between NBS subtypes and survival (log-rank test p-value < 0.05) (Table 2). Such significant associations were observed for certain gene panels and cluster numbers (Table 2). For example, when the UCEC samples were classified into 6 clusters using the TrueSeq panel, the clusters were well-separated, differed in survival (log-rank test p-value = 1.2e-6) and were balanced in cluster size (see Figure 2A-2B). Also with the TrueSeq panel, the CRC samples were classified into 3 clusters with significant association with survival (Figure 2C-D). Among the cancer types with survival data, only OV showed a significant association of subtypes with survival using the Full dataset (log-rank p-value 0.02) with K = 3. This is in contrast to the gene panels as listed in Table 2, under which significant (or marginally significant) associations between subtypes and survival were observed across multiple tumors and multiple K's (Figure S15-S17 in Additional file).

**Table 2:** Significant associations between NBS subtypes (clusters) and survival in 5 tumor types using 3 gene panels

Tumor	Panel	K	p-value
CRC	TruSeq	3	0.036
HNSC	TruSeq	4	0.02
KIRC	PanCan	3	0.02
LUAD	PanCan	3	1.1*10^-4^
UCEC	TruSeq	6	1.2*10^-6^

We then investigated the association between clusters and tumor grade/stage. Of the cancer types (HNSC, KIRC, OV, UCEC) with grade or stage information available, we found no association between NBS subtypes and tumor grade/stage except for UCEC. As shown in Figure 2, the subtypes of UCEC tumors with relatively poor survival (red, pink) were also enriched for high grade (p = 0.03, Fisher exact test).

For some tumor types, e.g. HNSC, KIRC and CRC, different *K *values generated nested subtypes (Fig. S15-S17 in Additional file). For example, for HNSC the yellow cluster for *K *= 3 was further divided into yellow and black clusters for *K *= 4, and the black cluster for *K *= 4 was further divided into black and pink clusters for *K *= 5, while all other clusters remain the same (Fig. S15 in Additional file). For larger *K *values, NBS classified patients into finer subgroups with distinct survival curves, and as a result the significance of association of survival with subtypes is consistent across different *K*'s (Fig. S15 in Additional file). Similar nested patterns were observed in KIRC and CRC (Fig. S16,S17 in Additional file).

**Figure 3:**
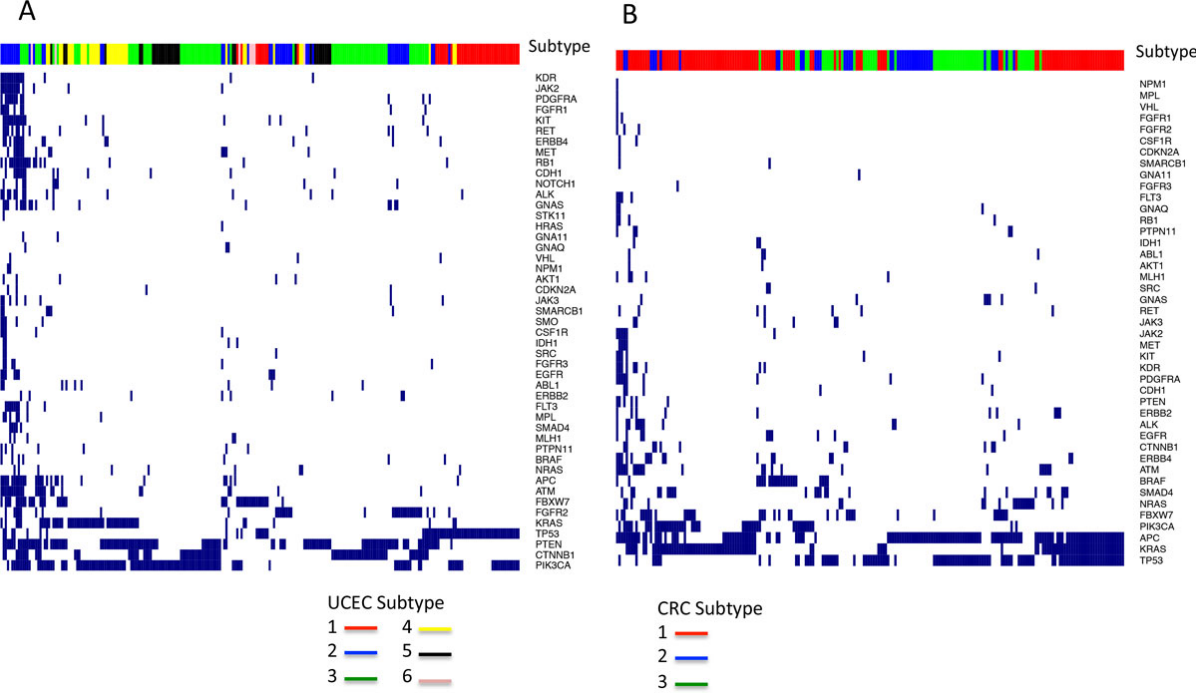
**Mutation profiles (before network smoothing) of UCEC (A) and CRC (B) based on the TrueSeq panel**.

We further examined the mutation patterns (before network smoothing) of UCEC and CRC that showed significant associations with survival using the TruSeq panel. In UCEC 6 subtypes were discovered. The cluster 2 (blue) has the best survival, and cluster 1 (red) and 6 (pink) are the worst (Figure 2A). The mutation patterns showed that cluster 2 harbors more mutations than other clusters (Figure 3A). It was observed that *POLE *is associated with hypermutations in UCEC [2] but this gene is absent in the TruSeq panel. We calculated the mutation frequency of *POLE *in these clusters and found that 32% of patients in cluster 2 have *POLE *mutations compared to 0-8% in other clusters, indicating that important mutation signatures can be captured by a panel of genes via NBS although the signature gene was not included in the panel. Cluster 1 (red) is the second worst survival cluster and a distinct signature is the high frequency of *TP53 *mutations compared to others (Figure 3A). Only 7 patients are in the worst cluster 6 (pink) and it is not clear what signatures are associated with this subtype due to the small size of the cluster. Overall, the clustering based on this small panel is highly consistent with that obtained previously, which was based on integrative analyses of multiple platforms [2]. For CRC, there are no clear mutation patterns in the gene panel (Figure 3B), and the separation of the better survival cluster (cluster 1, red, Figure 2C,D) from others may be due to complex combination of gene mutations linked through the biological network.

To investigate the effectiveness of traditional clustering approaches on mutation data, we applied hierarchical clustering on the mutation data without network smoothing of the 5 tumors that showed significant associations with survival. All of the tumors except UCEC failed to produce clear clustering, and essentially all samples were clustering into a single group (data not shown). Although UCEC showed separable subtypes, such subtypes are not associated with survival, indicating that clustering without NBS failed to capture clinically relevant pathways that are encoded in protein interaction networks.

Our results were consistent with the previous work in which NBS approach was applied to three cancers: OV, UCEC and LUAD [12]. Using the full exome-level functional mutation data, we observed classification of UCEC samples into 3 clusters with no significant difference in survival (log-rank test p-value = 0.63) (Fig. S14 in Additional file), similar to the result reported previously [12]. We also observed significant association between OV subtypes and survival (log-rank p = 0.02) (Fig. S14 in Additional file), although the significance levels are not the same, probably due to different samples used in the two studies (e.g ~1/3 samples are different due to differences in downloaded data).

## Discussion

Exome and whole genome sequencing have identified somatic mutations across multiple tumors; however most mutations are deemed passengers. Comprehensive analyses have pinpointed important genes and pathways that drive tumor progression. This enables the classification of patients into genetically distinct subtypes so that biologically driven prognostication and therapy selection become feasible in clinic. Network-based approaches integrate mutations and protein interaction networks to achieve knowledge-guided subtyping and have shown promises in linking subtypes with clinical data. In this study we showed that using a panel of important genes can achieve superior classification than using the full exome-level mutations, and often has better predictive performance of clinical outcome as well. Due to extensive tumor heterogeneity, however, it is infeasible to design a single panel to fit all cancers. The performance of a gene panel will likely depend on specific tumor types. We investigated three panels with different contents and numbers of genes in an effort to span a range of scenarios. For optimal performance it may warrant a focused investigation if a custom gene panel is to be designed for a specific tumor type.

NBS approaches leverage prior knowledge of protein interaction networks to overcome the challenge of the sparsity of somatic mutations. It is unclear, however, how to construct an optimal network used in NBS. It is expected that biological networks in different organs or tissues are different, and some genes in the network may not even be expressed in certain tissues. Other complicating factors in the NBS procedure involve various tuning parameters, such as the α in the network propagation step, the γ in the graph-regularization of the NMF. Together with the influence of the genes in panels, it is unclear how to obtain robust and consistent subtyping. For example, some tumor types have clear clustering of patients but the subtypes are not associated with clinical outcomes. For such cases it is also possible that clinical relevant tumor subtypes, if present, are driven by other mechanisms, such as methylation or copy number aberration, instead of somatic mutations. It is generally unknown *a priori *what strategy is optimal in tumor classification for clinic use, and more integrative analyses across large cohorts may provide further guidance towards this goal.

The motivation behind NBS is to deal with the sparsity of mutation data. For mutations that are frequently observed in multiple patients, it may be tempting to apply clustering methods on the sparse mutation data without network smoothing. Our results showed that even in subtypes that are enriched for specific mutations, e.g. UCEC, it is crucial to leverage prior knowledge encoded in gene networks to classify patients into clinically relevant subtypes. Gene interaction networks used in the NBS approach are not merely to deal with the sparsity of mutations but to provide meaningful biological knowledge to have effective subtyping. It may be true in general that NBS is beneficial for subtyping for other somatic mutation data and effectiveness of NBS in other scenarios needs further exploration.

## Conclusion

We applied NBS to 13 major cancer types using both exome-level mutation data and panels of genes for classification and showed that small panels are often more effective in clustering tumors than full exome data. In addition, subtypes discovered by panels of genes in 5 tumor types (CRC, HNSC, KIRC, LUAD and UCEC) are significantly associated with patient survival, while subtypes based on full exome mutations are less predictive of clinical data. Screening of panels of genes combined with the NBS analysis strategy has potential for clinical use and the performance may be further improved by selection of clinically important genes and proper gene interaction networks.

## Competing interests

The authors declare that they have no competing interests.

## Authors' contributions

XZ and BL designed the study and drafted the manuscript. XZ performed all the data analyses. SZ helped the preparation of figures and tables. HY and YS contributed to the writing of the manuscript. All authors read and approved the final manuscript.

## Supplementary Material

Additional file 1 **This file contains all supplementary Tables and Figures**. The file name is "Additional file" in PDF format. Click here for file

## Declarations

The publication costs for this article were funded by the last author.

This article has been published as part of *BMC Genomics *Volume 16 Supplement 7, 2015: Selected articles from The International Conference on Intelligent Biology and Medicine (ICIBM) 2014: Genomics. The full contents of the supplement are available online at http://www.biomedcentral.com/bmcgenomics/supplements/16/S7.
