# Supplementary material for: Network-based stratification analysis of 13 major cancer types using mutations in panels of cancer genes
Source: BMC Genomics. 2015 Jun 11;16(Suppl 7):S7. doi: 10.1186/1471-2164-16-S7-S7 (PMC4474538; doi:10.1186/1471-2164-16-S7-S7)
Supplement: Additional file 1 — This file contains all supplementary Tables and Figures. The file name is "Additional file" in PDF format. [file 1471-2164-16-S7-S7-S1.pdf]

**Table S1**: Sample sizes of 21 tumor types before and after filtering to ensure sufficient mutations per sample.

| Cancer[a] | Sample size | Full (# mutations ≥ 6) | FoundationOne (# mutations ≥ 1) | PanCan (# mutations ≥ 1) | TrueSeq (# mutations ≥1) |
|---|---|---|---|---|---|
| BLCA | 99 | 99 (100%) | 97(98%) | 95 (96%) | 84 (85%) |
| BRCA | 887 | 849 (96%) | 661 (75%) | 647 (73%) | 528 (60%) |
| CARC* | 54 | 53 (98%) | 26 (48%) | 13 (24%) | 10 (19%) |
| CLL* | 158 | 135 (85%) | 79 (50%) | 66 (42%) | 34 (22%) |
| CRC | 233 | 233 (100%) | 227 (97%) | 226 (97%) | 219 (94%) |
| DLBC* | 57 | 55 (96%) | 51 (89%) | 43 (75%) | 31 (54%) |
| ESO | 140 | 140 (100%) | 133 (95%) | 125 (89%) | 112 (80%) |
| GBM | 291 | 288 (99%) | 247 (85%) | 237 (81%) | 199 (68%) |
| HNSC | 384 | 372 (97%) | 357 (93%) | 347 (90%) | 303 (79%) |
| KIRC | 417 | 414 (99%) | 328 (79%) | 310 (74%) | 220 (53%) |
| LAML* | 194 | 126 (65%) | 132 (68%) | 131 (68%) | 77 (40%) |
| LUAD | 398 | 391 (98%) | 372 (93%) | 359 (90%) | 322 (81%) |
| LUSC | 176 | 176 (100%) | 176 (100%) | 175 (99%) | 158 (90%) |
| MED* | 89 | 42 (47%) | 26 (29%) | 24 (27%) | 15 (17%) |
| MEL | 118 | 118 (100%) | 117 (99%) | 113 (96%) | 112 (95%) |
| MM | 204 | 200 (98%) | 157 (77%) | 146 (72%) | 121(59%) |
| NB* | 75 | 18 (24.00%) | 49 (65%) | 61 (81%) | 62 (83%) |
| OV | 316 | 313 (99%) | 276 (87%) | 281 (89%) | 238 (75%) |
| PRAD* | 131 | 109 (83%) | 51 (39%) | 45 (34%) | 19 (15%) |
| RHAB* | 33 | 7 (21%) | 10 (30%) | 4 (12%) | 8 (24%) |
| UCEC | 247 | 247 (100%) | 245 (%99) | 242 (98%) | 229 (93%) |
| Total | 4701 | 4008[¶] | | | |

[*]Tumors excluded from NBS analysis due to insufficient mutations or samples
[¶]Calculated after excluding tumor marked by *
[a]BLCA-Bladder urothelial carcinoma; BRCA-Breast invasive carcinoma; CARC-Carcinoid; CLL-Chronic lymphocytic leukemia; CRC-Colorectal carcinoma; DLBC-Diffuse large B-cell lymphoma; ESO-Esophageal adenocarcinoma; GBM-Glioblastoma multiforme; HNSC-Head and neck squamous cell carcinoma; KIRC-Kidney renal clear cell carcinoma; LAML-Acute myeloid leukemia; LUAD-Lung adenocarcinoma; LUSC-Lung squamous cell carcinoma; MED-Medulloblastoma; MEL-Melanoma; MM-Multiple myeloma; OV-Ovarian serous cystadenocarcinoma; PRAD-Prostate adenocarcinoma; RHAB-Rhabdoid tumor; UCEC-Uterine corpus endometrial carcinoma

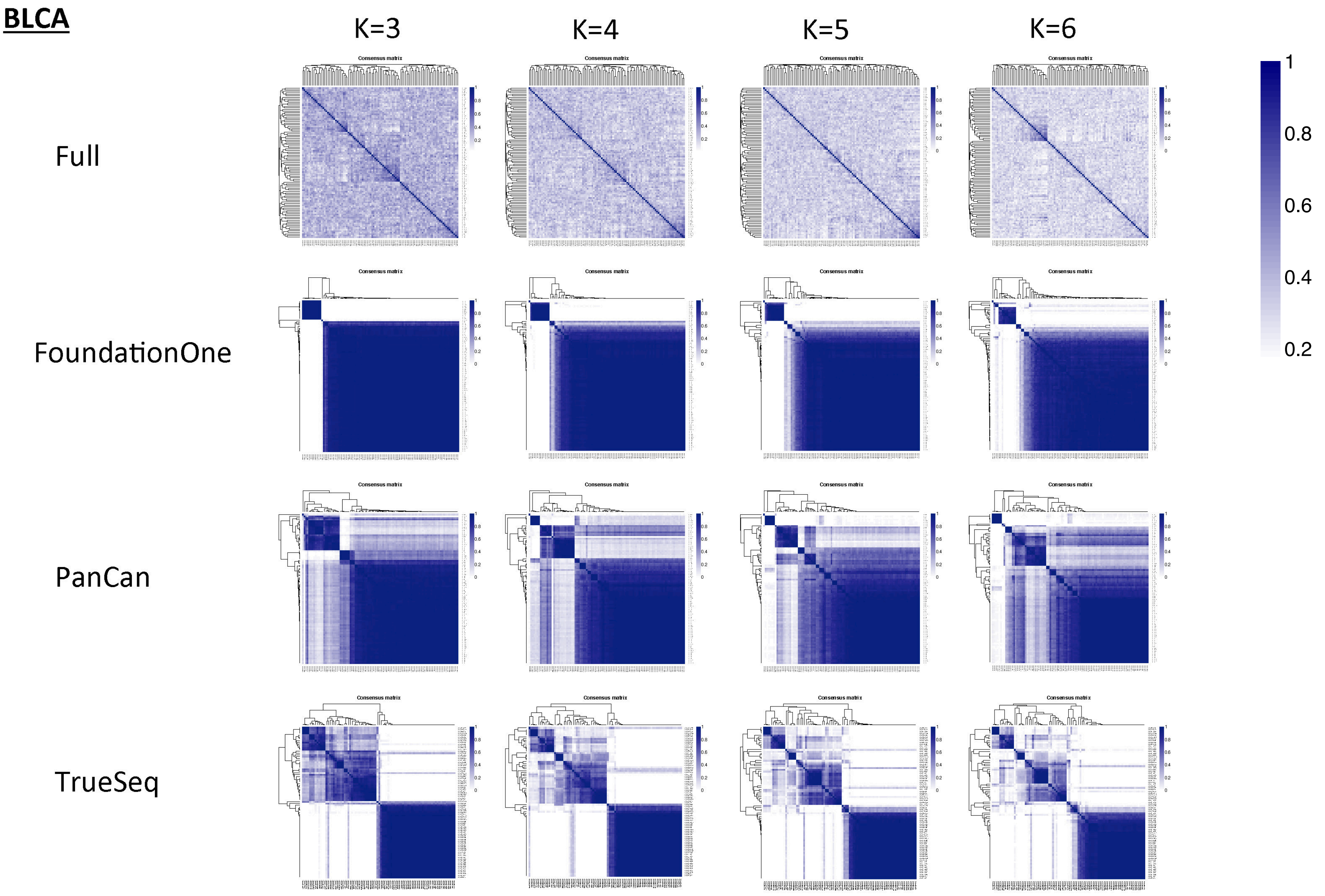


**Figure S1**: The clustering patterns of BLCA across the four gene sets (Full, FoundationOne, PanCan, and TrueSeq) and rank *K*=3,4,5,6.

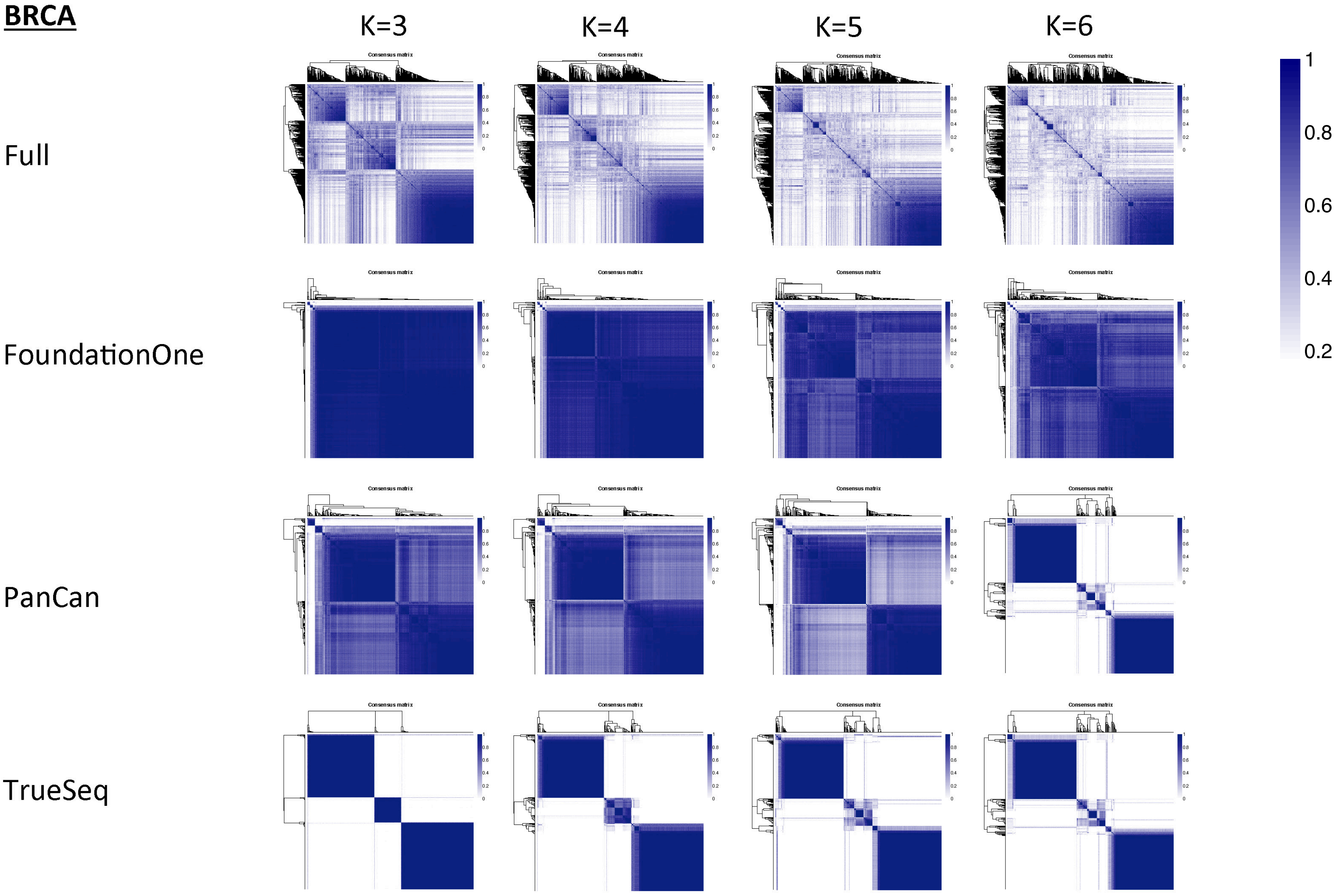


**Figure S2**: The clustering patterns of BRCA across the four gene sets (Full, FoundationOne, PanCan, and TrueSeq) and rank *K*=3,4,5,6.

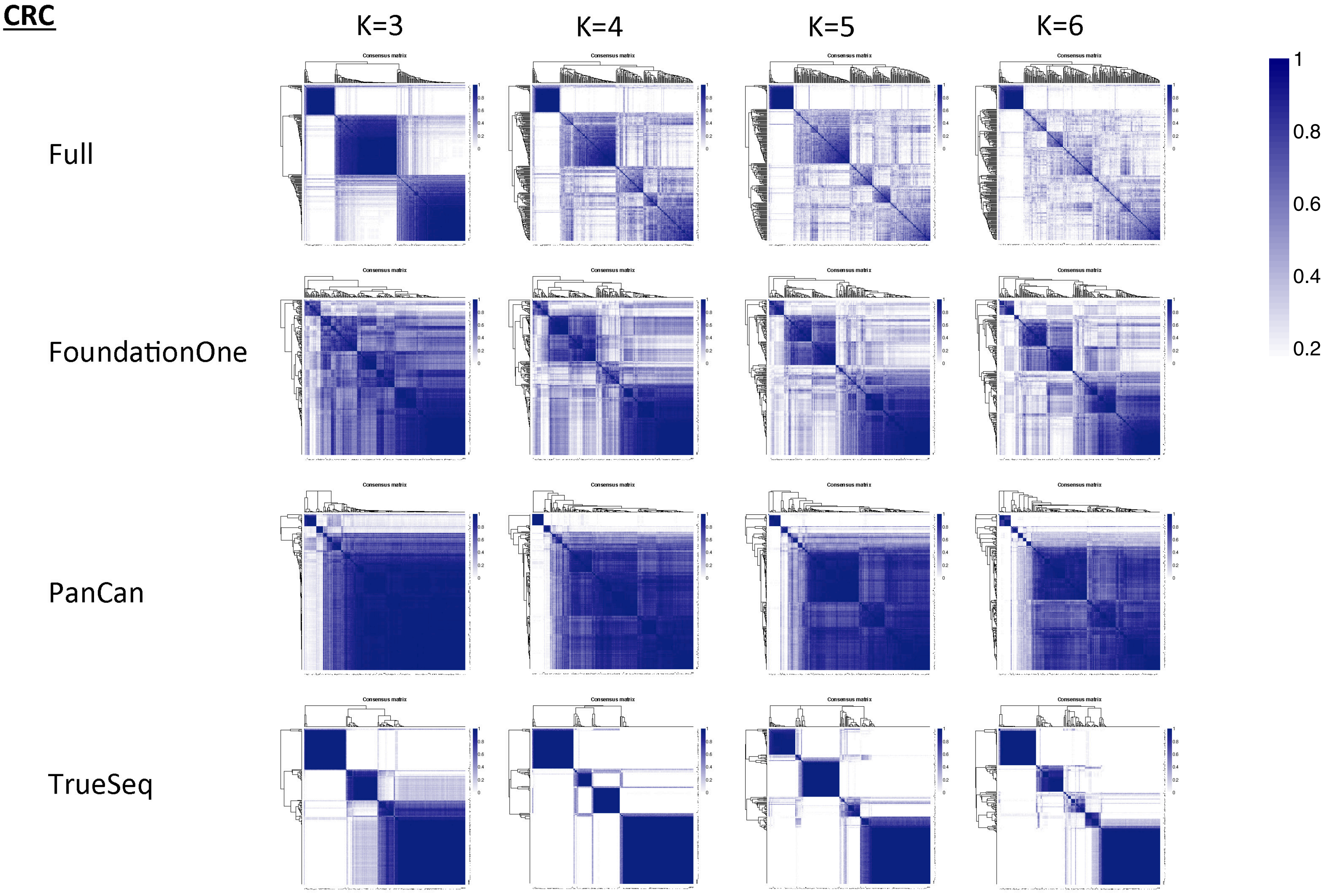


**Figure S3**: The clustering patterns of CRC across the four gene sets (Full, FoundationOne, PanCan, and TrueSeq) and rank *K*=3,4,5,6.

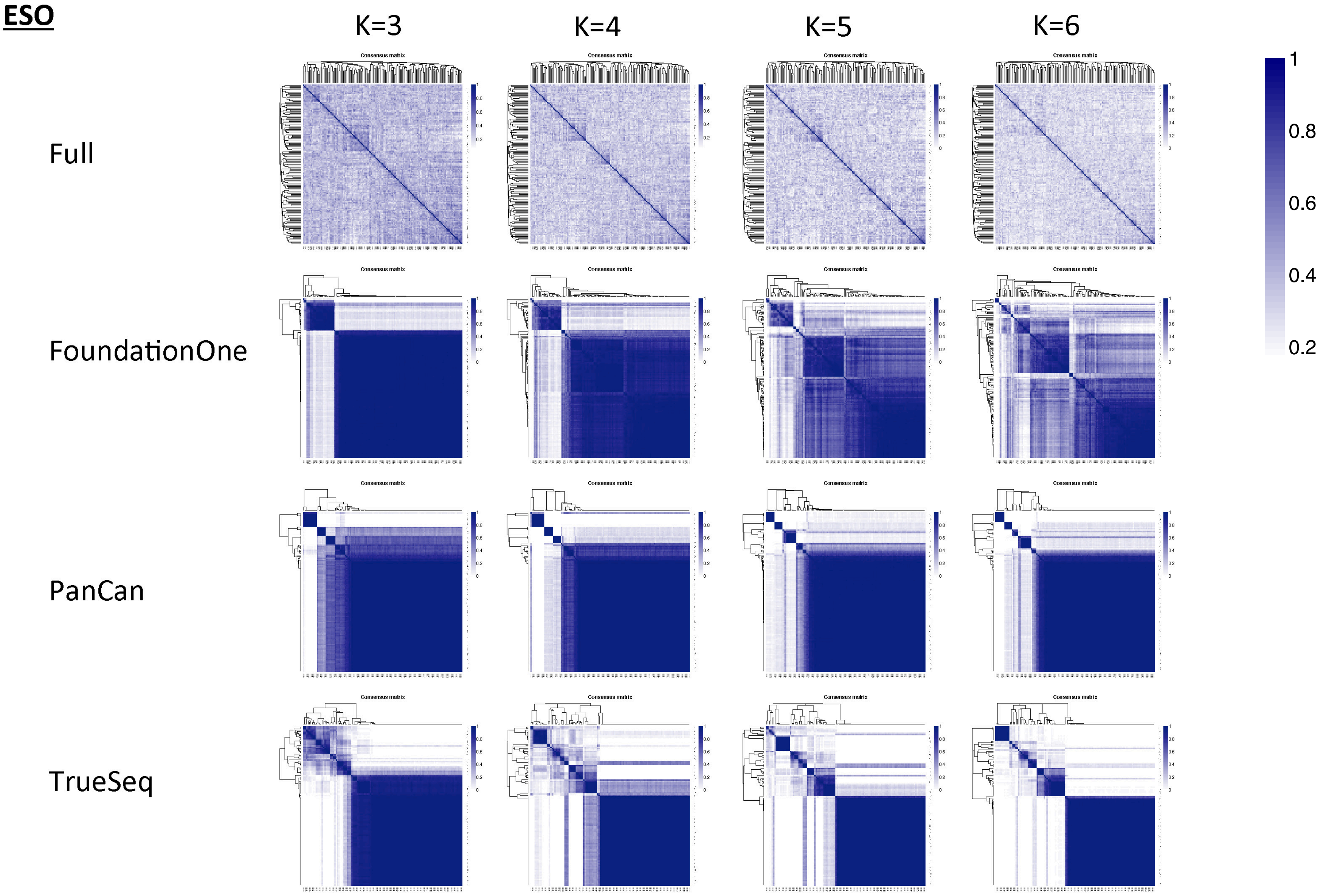


**Figure S4**: The clustering patterns of ESO across the four gene sets (Full, FoundationOne, PanCan, and TrueSeq) and rank *K*=3,4,5,6.

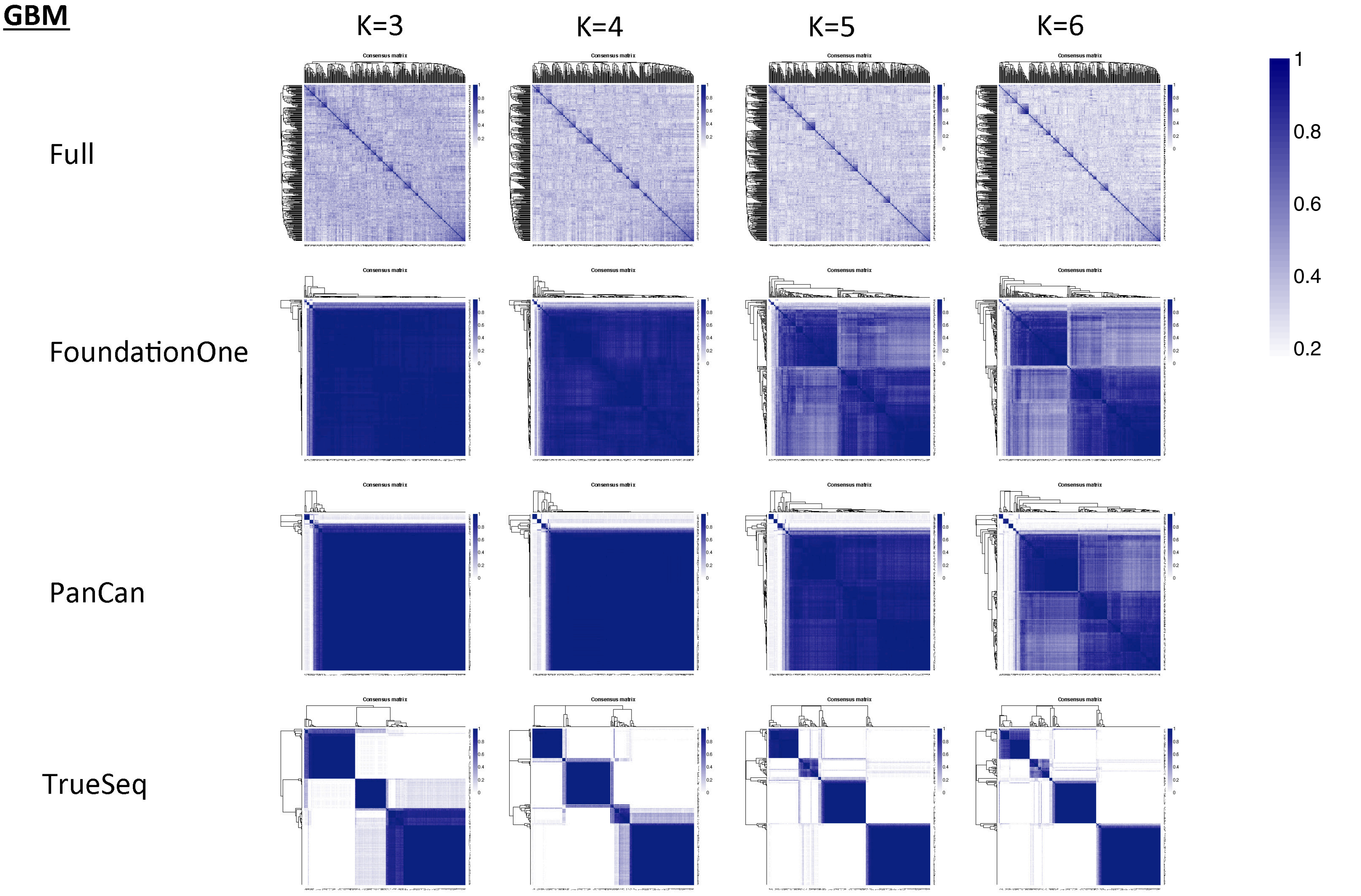


**Figure S5**: The clustering patterns of GBM across the four gene sets (Full, FoundationOne, PanCan, and TrueSeq) and rank *K*=3,4,5,6.

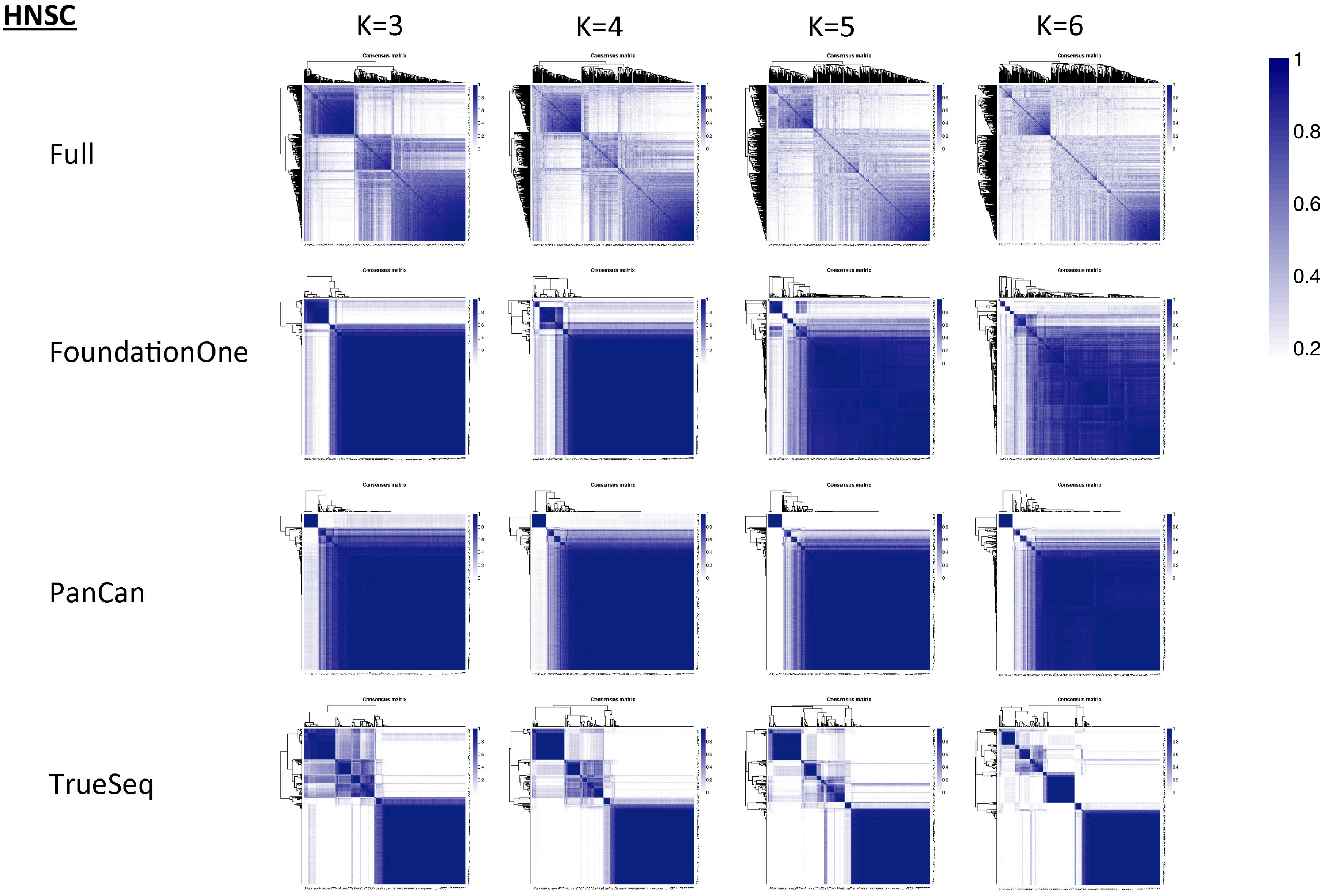


**Figure S6**: The clustering patterns of HNSC across the four gene sets (Full, FoundationOne, PanCan, and TrueSeq) and rank *K*=3,4,5,6.

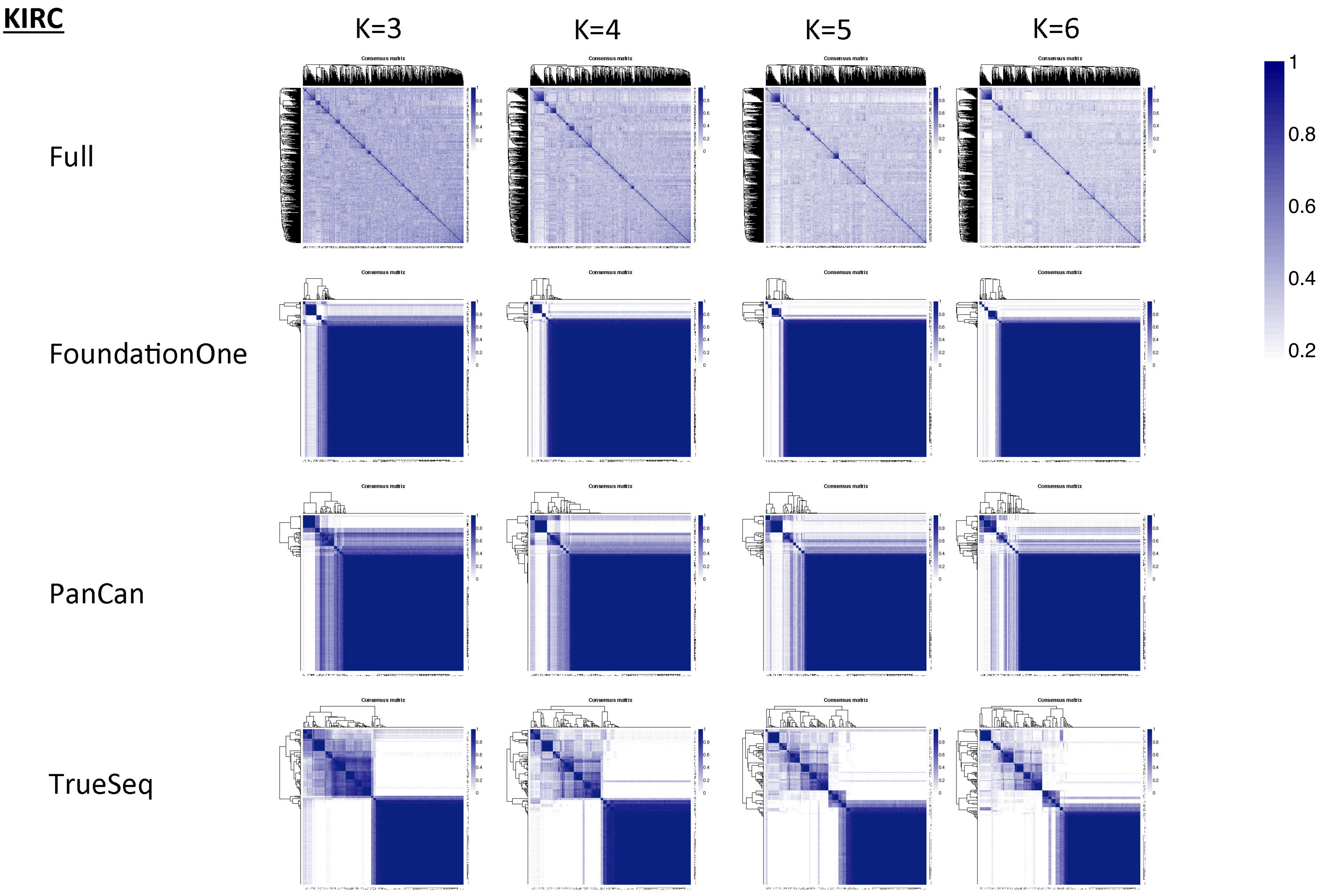


**Figure S7**: The clustering patterns of KIRC across the four gene sets (Full, FoundationOne, PanCan, and TrueSeq) and rank *K*=3,4,5,6.

**LUAD**

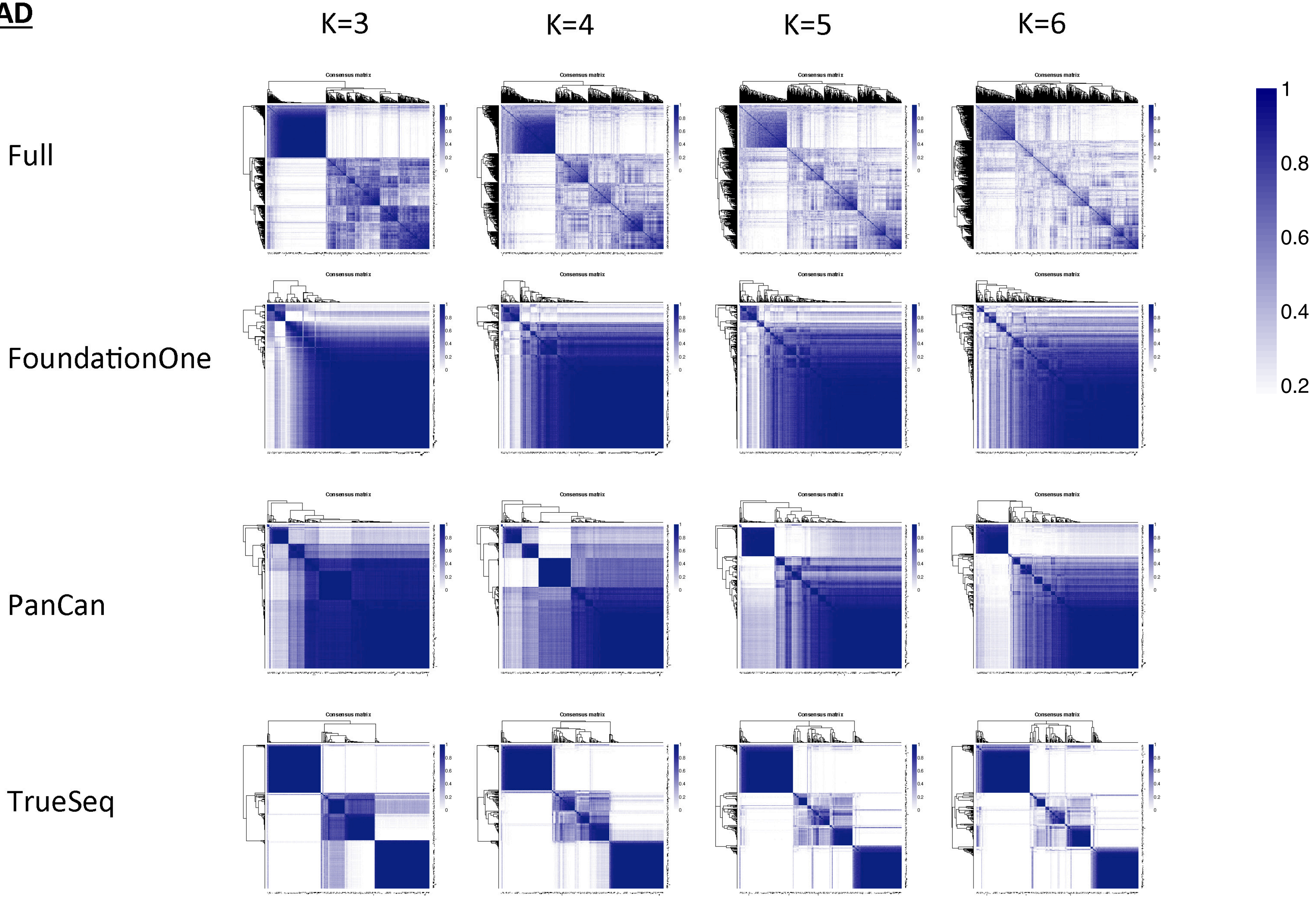


**Figure S8**: The clustering patterns of LUAD across the four gene sets (Full, FoundationOne, PanCan, and TrueSeq) and rank *K*=3,4,5,6.

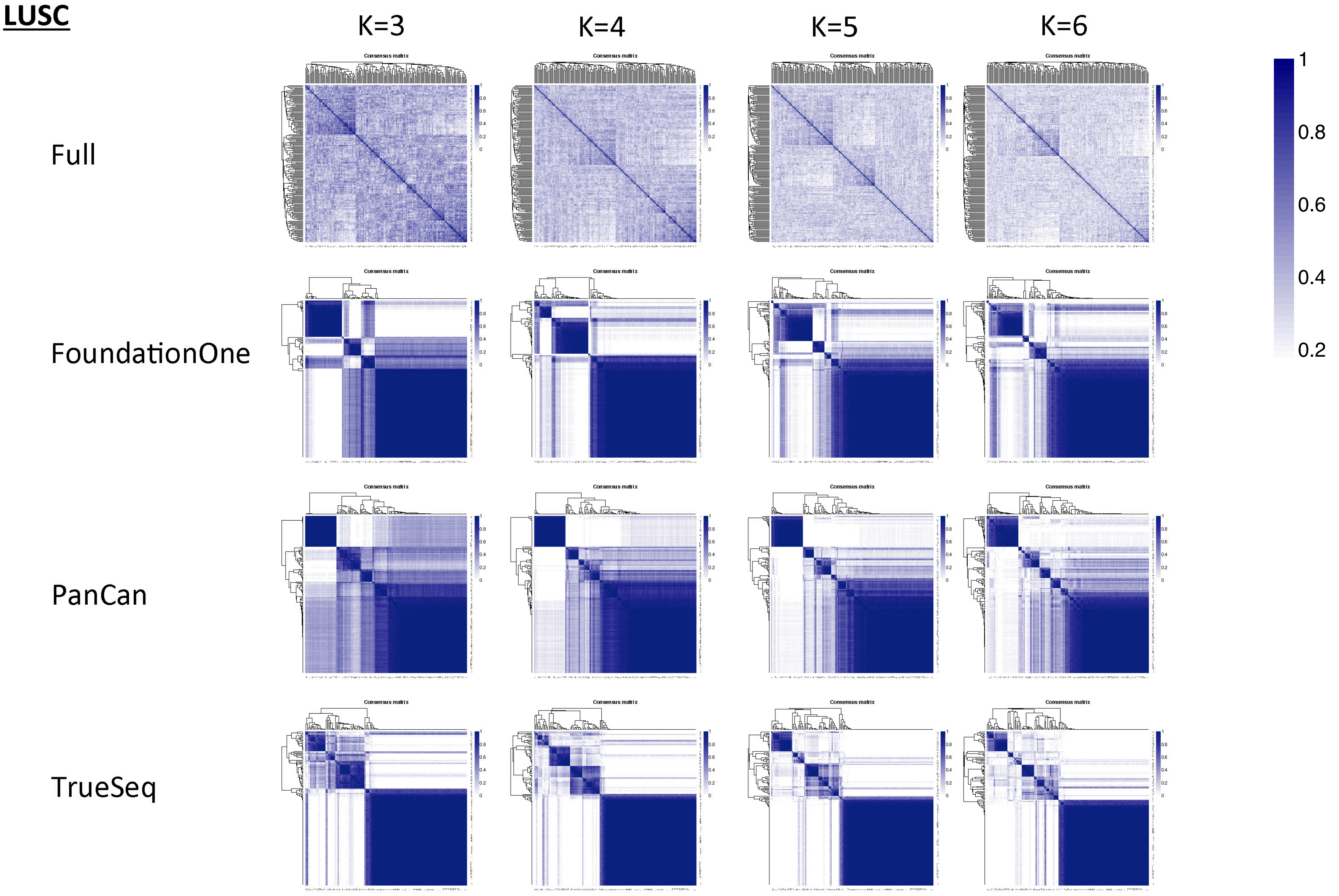


**Figure S9**: The clustering patterns of LUSC across the four gene sets (Full, FoundationOne, PanCan, and TrueSeq) and rank *K*=3,4,5,6.

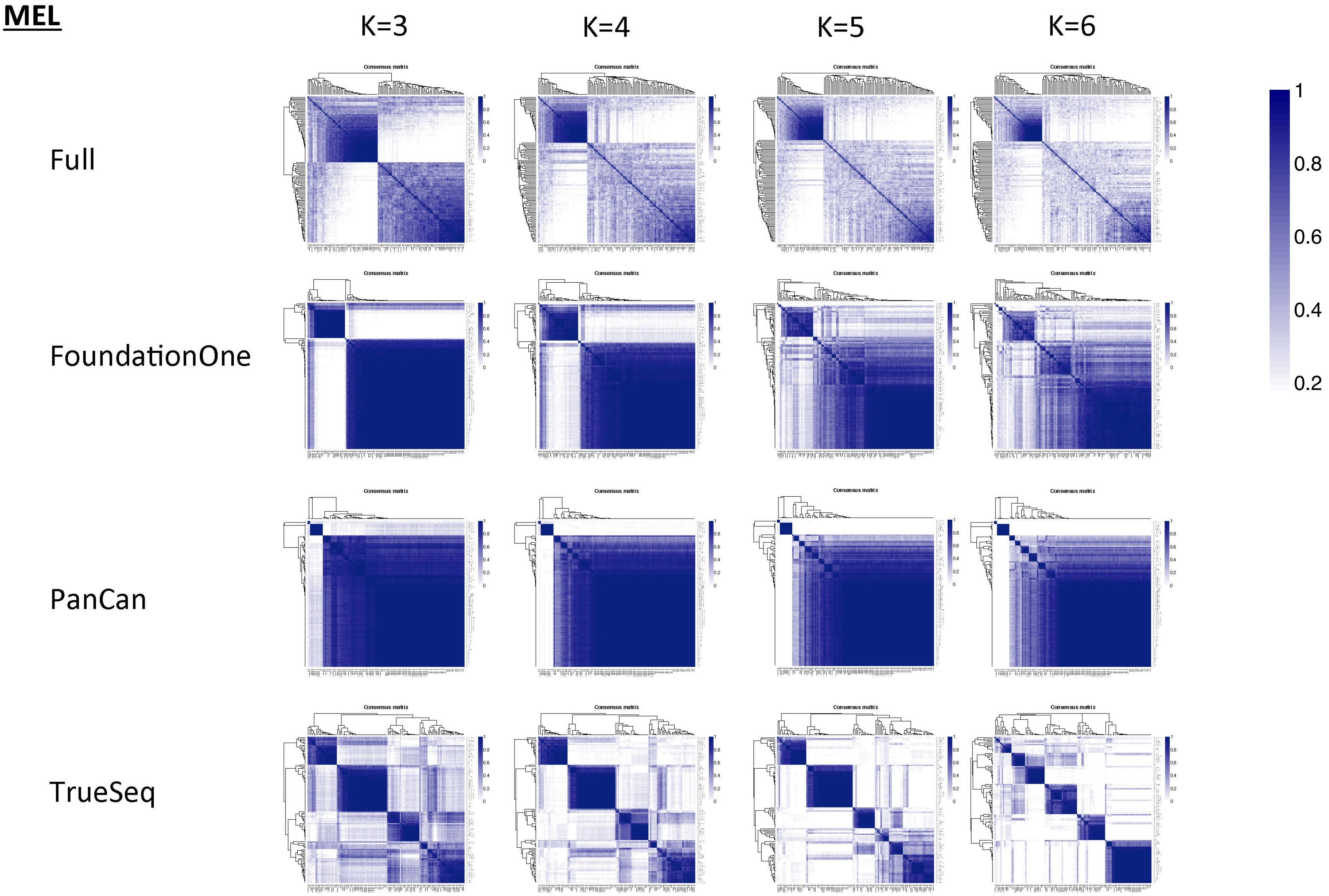


**Figure S10**: The clustering patterns of MEL across the four gene sets (Full, FoundationOne, PanCan, and TrueSeq) and rank *K*=3,4,5,6.

## MM

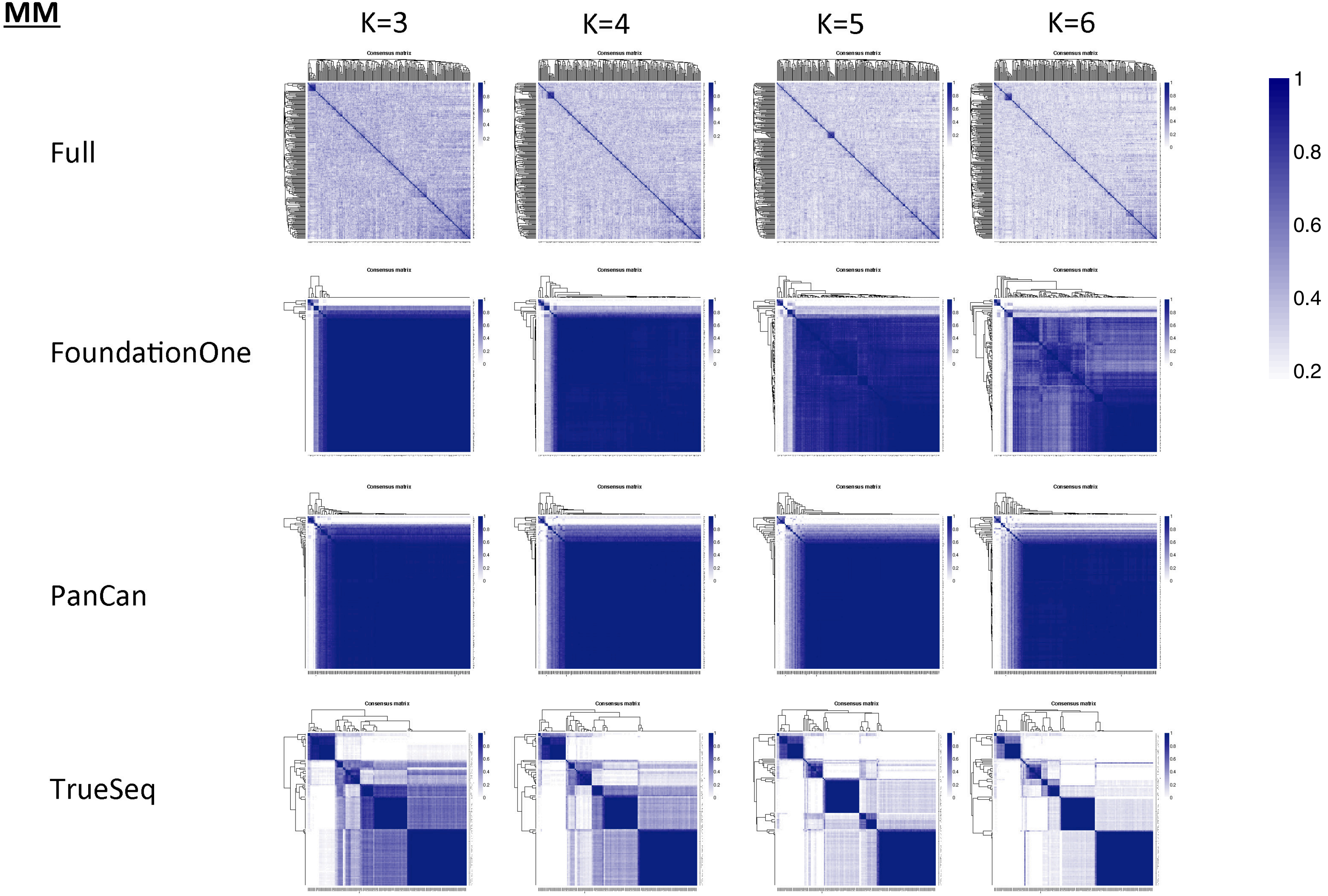


**Figure S11**: The clustering patterns of MM across the four gene sets (Full, FoundationOne, PanCan, and TrueSeq) and rank *K*=3,4,5,6.

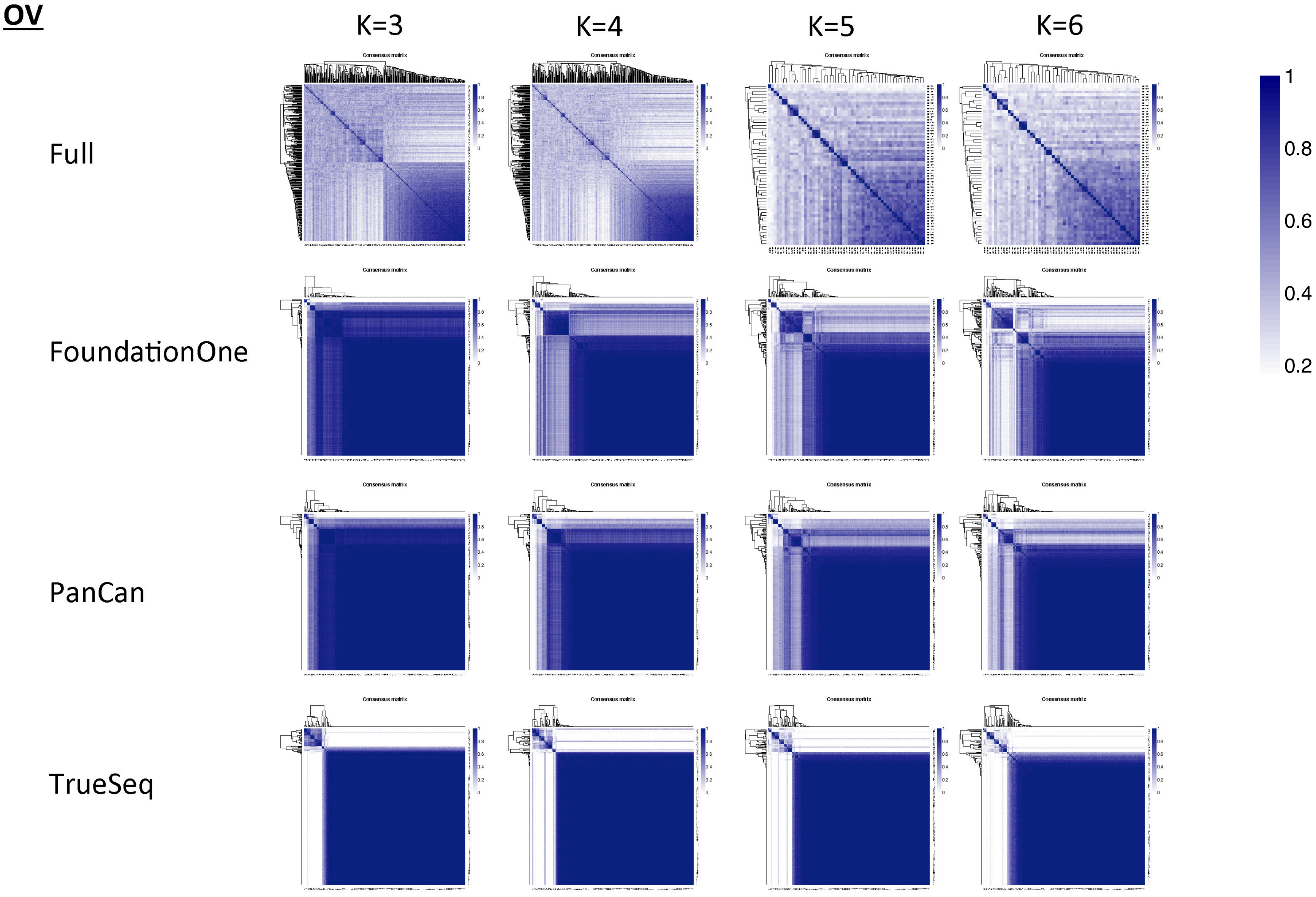


**Figure S12**: The clustering patterns of OV across the four gene sets (Full, FoundationOne, PanCan, and TrueSeq) and rank *K*=3,4,5,6.

## UCEC

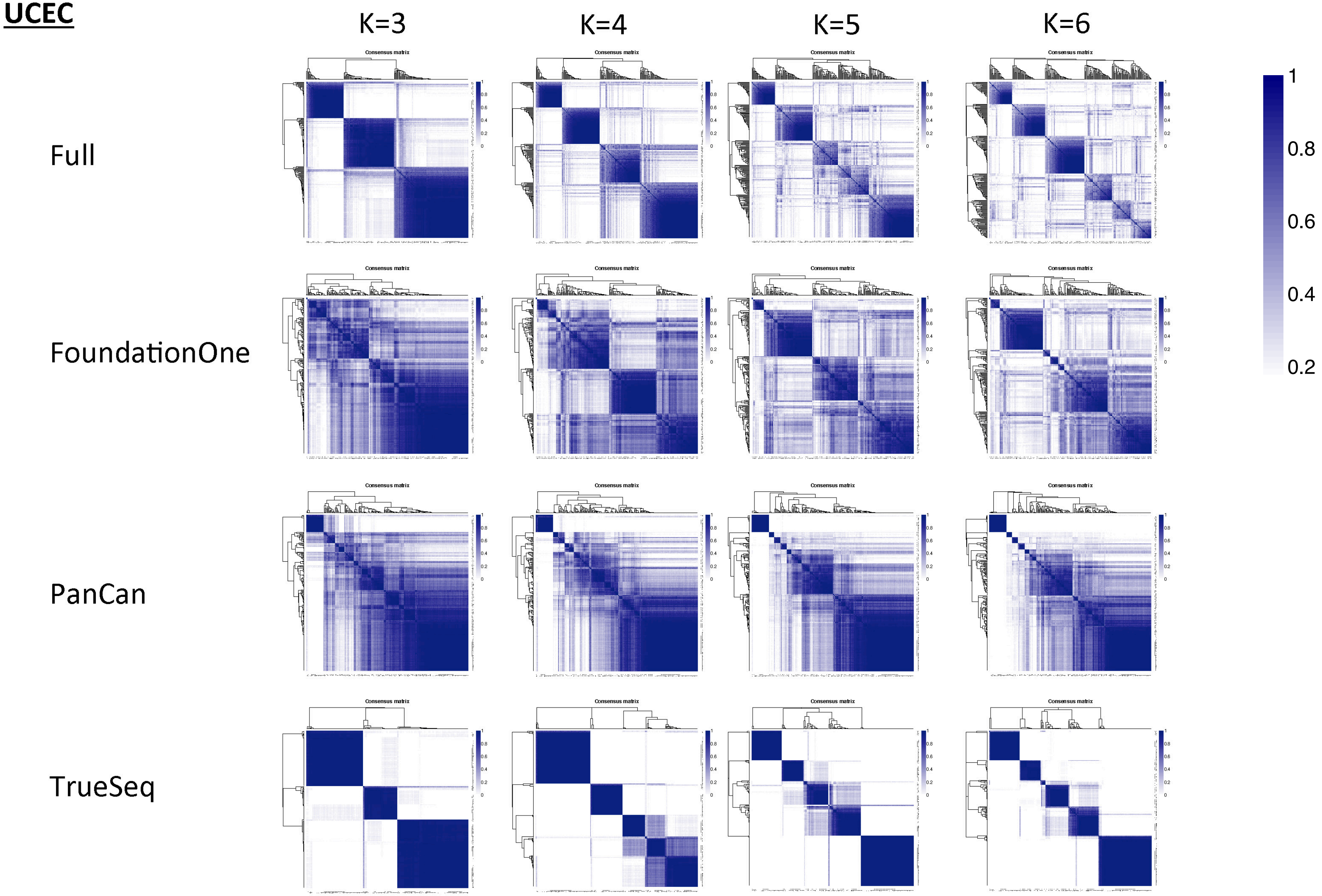


**Figure S13**: The clustering patterns of UCEC across the four gene sets (Full, FoundationOne, PanCan, and TrueSeq) and rank *K*=3,4,5,6.

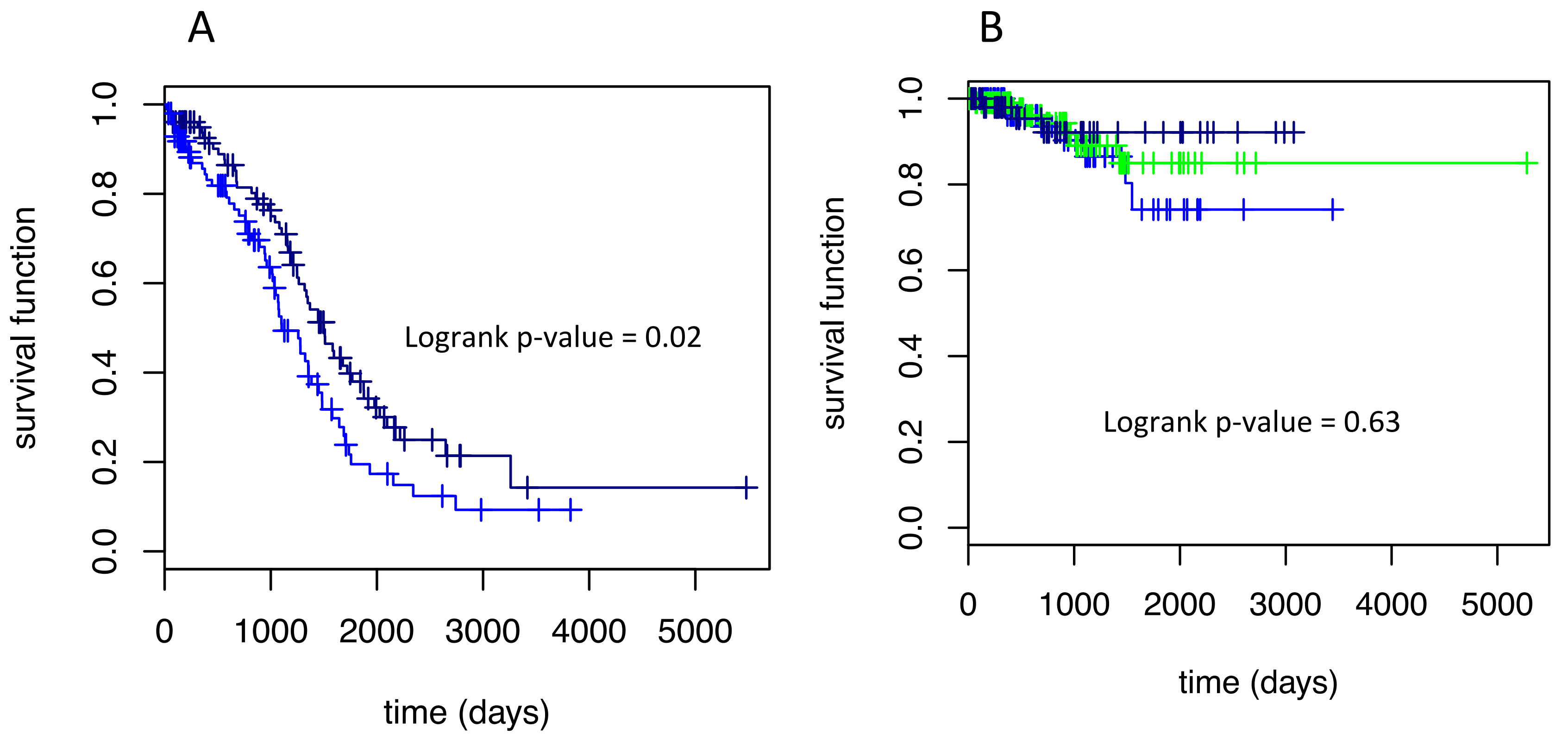


**Figure S14**: NBS subtypes using the Full dataset and the associations with survival for three cancers, OV (A) and UCEC (B).

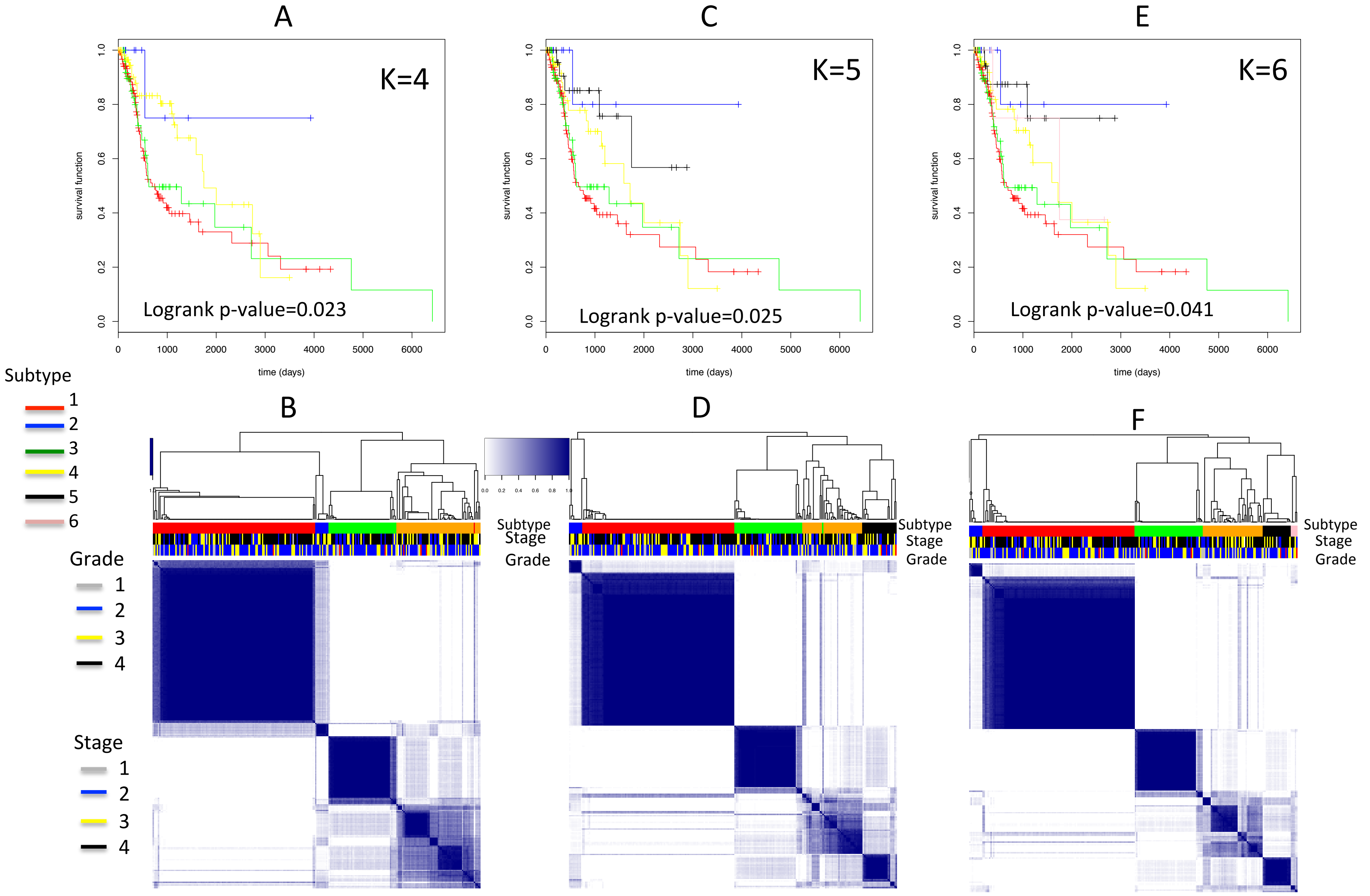


**Figure S15**: Subtypes associated with survival across different rank *K*'s for HNSC based on the TrueSeq panel. The sample subtype assignments for K=4,5,6, display an approximate nesting structure.

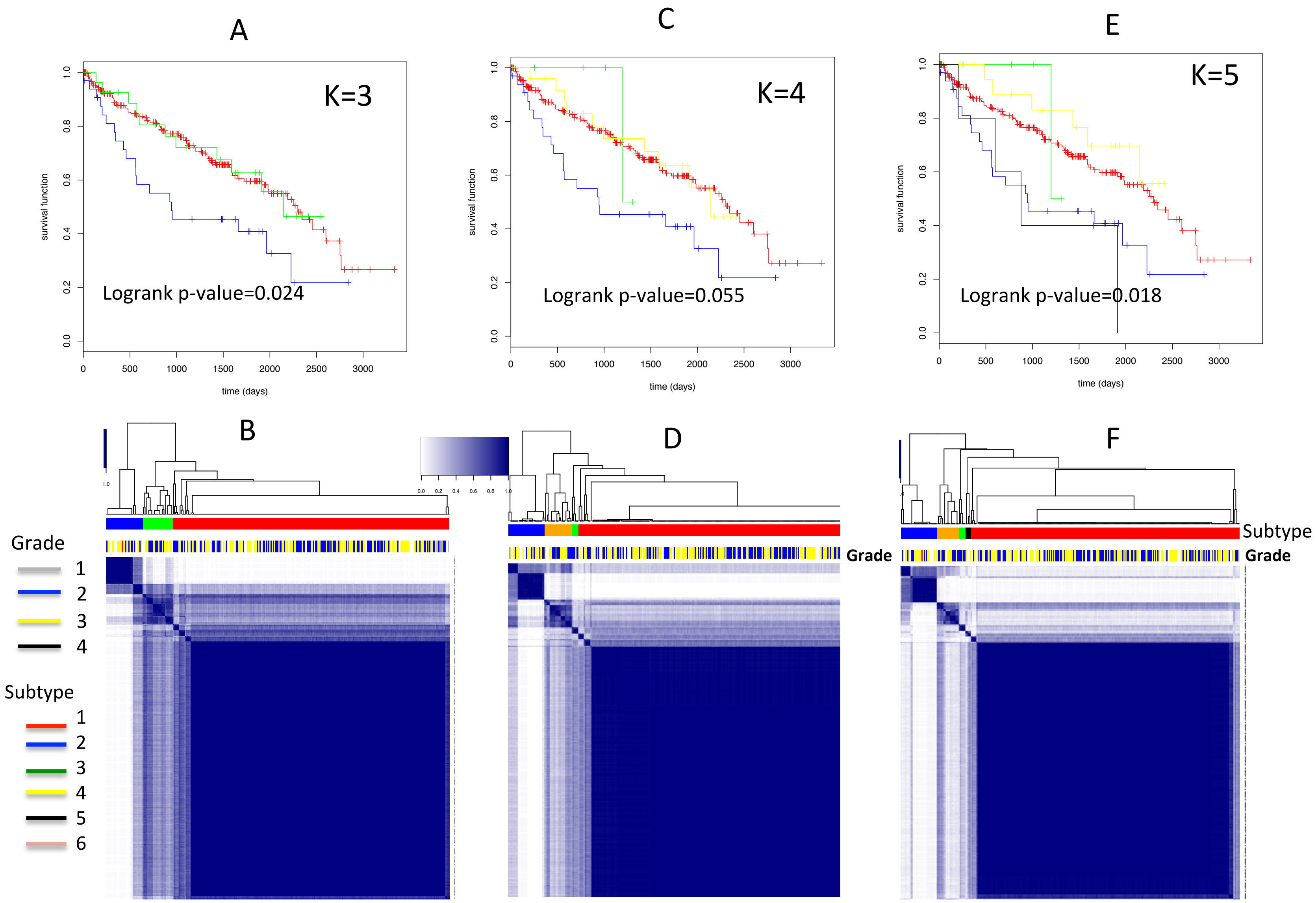


**Figure S16:** Subtypes associated with survival across different rank *K*'s for KIRC based on the PanCan panel. The sample subtype assignments for K=3,4,5, display an approximate nesting structure.

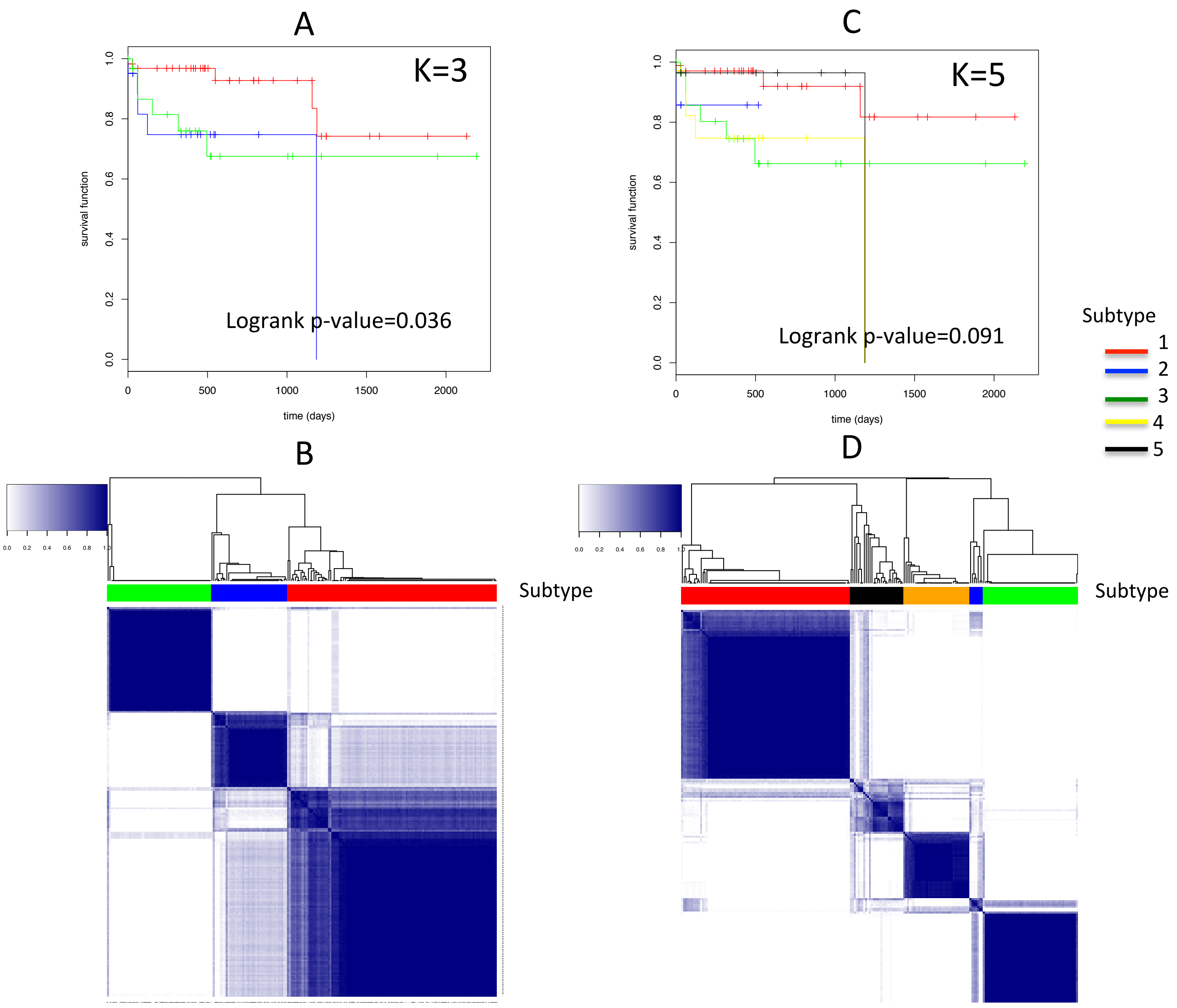


**Figure S17:** Subtypes associated with survival across different rank *K*'s for CRC based on the TrueSeq panel. The sample subtype assignments for K=3,5, display an approximate nesting structure.
